# Optimizing catheter-directed thrombolysis access and predicting post-thrombotic syndrome in acute entire-limb DVT: a retrospective cohort study and nomogram development

**DOI:** 10.3389/fcvm.2026.1839821

**Published:** 2026-05-13

**Authors:** Cheng Qian, Guo-Ping Chen, Tao Wang, Wen-Sheng Lou, Jian-Ping Gu

**Affiliations:** Department of Interventional Radiology, Nanjing First Hospital, Nanjing Medical University, Nanjing, China

**Keywords:** catheter-directed thrombolysis, deep vein thrombosis, post-thrombotic syndrome, risk factors, venous access

## Abstract

**Background:**

Selecting the optimal catheter-directed thrombolysis (CDT) access for acute entire-limb deep vein thrombosis (DVT) remains controversial. This study aimed to compare the technical and clinical outcomes of three CDT access approaches and to develop a prognostic nomogram for predicting post-thrombotic syndrome (PTS) based on hemodynamic and procedural variables.

**Methods:**

We retrospectively analyzed 172 patients with acute entire-limb DVT who underwent CDT via contralateral femoral vein access (CFVA, *n* = 87), ipsilateral popliteal vein access (IPVA, *n* = 34), or ipsilateral calf venous access (ICVA, *n* = 51). Procedural metrics, angiographic patency, and 2-year PTS incidence were evaluated. A LASSO-Cox regression model was utilized to address multicollinearity, identify independent PTS predictors, and construct a prognostic nomogram.

**Results:**

Although CFVA afforded the shortest sheath insertion time (1.74 ± 1.02 min), it required significantly longer access establishment (17.07 ± 6.81 min) than IPVA and ICVA (*P* < 0.001). Antegrade approaches (IPVA and ICVA) yielded superior femoropopliteal thrombus clearance and enhanced inflow patency compared to CFVA (*P* < 0.001). The 2-year PTS incidence was significantly higher in the CFVA group (59.8%) versus the IPVA (38.2%) and ICVA (41.2%) cohorts (*P* = 0.033). LASSO-Cox analysis identified poor inflow patency (HR 3.19), poor outflow patency (HR 2.17), and deep femoral vein axial transformation (HR 2.44) as independent risk factors for PTS. Prolonged thrombolysis duration and subsequent endovascular interventions were protective. The developed nomogram demonstrated excellent discriminative ability (C-index = 0.82).

**Conclusion:**

For acute entire-limb DVT, antegrade CDT access optimizes early femoropopliteal thrombus resolution. However, long-term PTS prevention is fundamentally driven by comprehensive hemodynamic restoration rather than the initial access site alone. Our proposed nomogram serves as a robust, evidence-based tool for individualized risk stratification and clinical decision-making.

## Introduction

Deep vein thrombosis (DVT) remains a leading cause of morbidity within the spectrum of venous thromboembolism. Although anticoagulation forms the cornerstone of management, catheter-directed thrombolysis (CDT) has emerged as a vital therapeutic option for patients with extensive iliofemoral or entire-limb thrombosis. The primary goal of CDT is to rapidly restore venous patency, preserve valvular function, and ultimately mitigate the debilitating long-term sequelae of post-thrombotic syndrome (PTS) (1). While earlier trials and registry data demonstrated that CDT accelerates symptom relief and promotes recanalization (2–4), its definitive impact on PTS prevention remains a subject of intense debate, particularly after the ATTRACT trial reported no overall reduction in PTS compared with standard anticoagulation (5).

This controversy underscores the importance of the “open-vein” hypothesis, which posits that effective early thrombus clearance—particularly within the femoropopliteal segment—is essential for re-establishing robust inflow to the common femoral vein. This hemodynamic restoration is a critical determinant of long-term venous patency and a primary defense against PTS. While pharmacomechanical CDT (PCDT) facilitates rapid initial clot removal, it has not consistently yielded superior long-term PTS prevention compared to standard CDT (6–10). Consequently, standard CDT continues to be widely utilized, especially in Asia, where “entire-limb DVT”—characterized by a massive thrombus burden extending continuously from the calf veins to the iliofemoral segment—is highly prevalent (11). However, in such extensive thrombotic scenarios, the optimal vascular access route to maximize thrombolytic efficacy remains poorly defined (4, 11–13).

Currently, several venous access strategies are employed, primarily categorized into antegrade approaches [ipsilateral popliteal vein access (IPVA) and ipsilateral calf venous access (ICVA)] and the retrograde approach [contralateral femoral vein access (CFVA)] (14). Each route presents distinct anatomical and hemodynamic trade-offs. Antegrade access (IPVA/ICVA) aligns with the natural direction of venous return, potentially optimizing the pharmacokinetic distribution of thrombolytic agents and enhancing direct clot dissolution in the crucial femoropopliteal segment. Conversely, while CFVA is technically straightforward due to consistent proximal anatomy, its retrograde catheter navigation against venous valves may result in suboptimal distal thrombus exposure.

Given the inconsistent conclusions of prior studies and the lack of robust evidence specific to entire-limb DVT, a comprehensive evaluation is urgently needed. Therefore, this study aimed to not only compare the technical efficacy and 2-year clinical outcomes of the three CDT approaches (CFVA, IPVA, and ICVA) but also to identify independent risk factors for PTS. Furthermore, utilizing LASSO-Cox regression to handle complex clinical variables, we sought to develop and validate a prognostic nomogram, thereby providing a practical, evidence-based tool to assist clinicians in optimizing personalized management strategies for patients with entire-limb DVT.

## Materials and methods

### Study design

This single-center retrospective cohort study included patients diagnosed with acute entire-limb DVT who underwent CDT between January 2018 and December 2022. Entire-limb DVT was defined as thrombus extending from the calf deep veins to the iliac venous system, confirmed by duplex ultrasonography or venography.

Inclusion criteria were: (1) age 18–75 years; (2) symptoms duration <14 days, (3) unilateral entire-limb DVT; and (4) at least 12 months of follow-up. Exclusion criteria included: (1) bilateral DVT; (2) previous ipsilateral DVT; (3) thrombus extension into the inferior vena cava (IVC); (4) history of pelvic malignancy; (5) concomitant pharmacomechanical thrombectomy. The institutional review board approved this study and waived informed consent due to its retrospective nature.

### CDT procedures

All patients received initial anticoagulation with low-molecular-weight heparin. Procedures were performed under local anesthesia by two interventional radiologists with 3–5 years of experience. Access selection depended on venous anatomy and operator judgment: (1) Contralateral femoral vein access (CFVA): Utilized when severe ipsilateral lower limb edema that compromised accurate puncture of the popliteal or calf vein; ipsilateral popliteal or calf veins exhibited stenosis, tortuosity, or anatomic variations that hindered safe cannulation. (2) Ipsilateral popliteal vein access (IPVA) and ipsilateral calf venous access (ICVA): Preferred when antegrade navigation was feasible and advantageous for thrombus traversal. An IVC filter (OptEase, Cordis, USA) was routinely implanted via CFVA prior to CDT initiation (15).

### Catheterization and thrombolysis

For CFVA, a Simon-1 or Cobra catheter with a 0.035-inch guidewire was advanced retrograde toward the distal calf veins. For IPVA/ICVA, venous puncture under venographic guidance was followed by antegrade catheter advancement to the iliac veins using a VER catheter.

A multiside-hole infusion catheter (Uni*Fuse, USA) was positioned across the thrombus. Urokinase was infused at 2 × 10^4^ U/h (maximum 50 × 10^4^ U/day) for up to 7 days. Daily venography assessed thrombus progression. Thrombolysis was modified or paused based on fibrinogen levels (<1.5 g/L: dose reduction by 50%; <1.0 g/L or if any clinical signs of major bleeding occurred: cessation) (16).

Residual iliac stenosis >50% was treated using balloon angioplasty (10 mm–12 mm) and stenting (12 mm–16 mm, Wallstent). The IVC filter was removed following thrombolysis.

Patients received oral anticoagulation (warfarin with INR 2–3 or rivaroxaban) for ≥3 months and wore compression stockings for ≥6 months.

### Technical evaluation and definitions

Sheath insertion time: From local anesthesia to successful sheath placement. Catheterization time: From initial attempt to cross the thrombus to final infusion catheter placement.

Venography images were interpreted by three radiologists, each with over 5 years of experience. Thrombolysis efficiency was evaluated by a modified SVS scoring system. The clot burden reduction rate was calculated as follows: (pre-CDT scores − post-CDT scores)/pre-CDT scores × 100%, and was categorized into three grades: Grade I for less than 50% reduction; Grade II for a 50%–95% reduction; and Grade III for 95%–100% reduction. Grade II/III defined as effective (12). The degree of inflow and outflow patency was categorized as follows: “Good” patency was defined as the trunk of the veins being detectable. Complete un-detection of the trunk was considered as “Poor” patency. Deep femoral vein axial transformation was evaluated according to established imaging criteria (17).

### Follow-up and outcome measures

Patients were evaluated at 1, 3, 6, 12 months, and annually. PTS incidence and severity were scored via the Villalta scale (18). Quality of life was evaluated with VEINES-QOL/Sym (19).

The primary endpoint was the PTS incidence. Secondary outcomes included technical indicators, thrombolysis duration, urokinase dosage, venous patency, need for subsequent interventions, segmental thrombolytic efficacy, and procedural complications.

### Statistical analysis

Continuous variables were reported as mean ± standard deviation and compared using one-way ANOVA. Categorical variables were compared using chi-square or Fisher's exact tests. These statistical analyses were performed using SPSS software (version 23.0). Kaplan–Meier curves and log-rank testing assessed PTS-free survival. LASSO Cox regression identified key predictors of PTS. All patients were followed for a minimum of 12 months. For the 36 patients who did not reach the 24-month endpoint, data were treated as right-censored at the time of last contact. Significant factors were incorporated into a multivariate Cox model, and a nomogram was generated using R software (version 4.3.1). Heatmap visualization was performed in Python (version 3.9). A two-sided *P* < 0.05 indicated statistical significance.

## Results

### Baseline characteristics

The cohort comprised 172 patients diagnosed with acute entire-limb DVT, stratified into the ICVA (*n* = 51), IPVA (*n* = 34), and CFVA (*n* = 87) access groups. The mean age of the cohort was 56.3 ± 12.1 years, with a male proportion of 39.5%. Baseline demographics, underlying risk factors, and symptom duration were well-balanced across all three cohorts, exhibiting no statistically significant differences (Table 1).

**Table 1:** The baseline characteristics of the patients with entire-limb deep venous thrombosis.

Characteristics	ICVA (*N* = 51)	IPVA (*N* = 34)	CFVA (*N* = 87)	*p* Value
Age, years	56.69 (11.27)	54.03 (13.50)	56.89 (12.00)	0.486
Gender, male	21 (41.2)	16 (47.1)	31 (35.6)	0.492
Onset time, days	5.56 (4.02)	6.10 (4.34)	6.37 (3.85)	0.519
Affected limb, left	30 (58.8)	20 (58.8)	54 (62.1)	0.910
Risk factors				
May-Thurner syndrome	34 (66.7)	21 (61.8)	54 (62.1)	0.844
Cancer	6 (11.8)	3 (8.8)	9 (10.3)	0.909
Recent surgery	12 (23.5)	7 (20.6)	17 (19.5)	0.855
Immobilization	7 (13.7)	5 (14.7)	10 (11.5)	0.868
Other hypercoagulable state^a^	2 (3.9)	1 (2.9)	4 (4.6)	0.916

Data presented as mean (SD) or No. (%).

a Other hypercoagulable state, such as nephrotic syndrome, anti-phospholipid syndrome and so on.

### Procedural performance

Procedural metrics exhibited profound access-dependent variations (Table 2). Although the CFVA approach afforded the most rapid initial sheath insertion (1.74 ± 1.02 min), it paradoxically required a significantly prolonged overall access establishment time (17.07 ± 6.81 min) compared to the IPVA (2.44 ± 0.66 min) and ICVA (2.63 ± 0.75 min) routes (*P* < 0.001). Furthermore, the CFVA group necessitated the longest thrombolysis duration (7.65 ± 1.15 days) and the highest cumulative urokinase dosage (375.63 ± 51.52 × 10^4^ U). Conversely, ICVA yielded the shortest infusion period (6.39 ± 1.50 days) and minimal dosage.

**Table 2:** CDT technical indicators.

Characteristics	ICVA (*N* = 51)	IPVA (*N* = 34)	CFVA (*N* = 87)	*p* Value
Sheath time (min)	9.02 (2.88)^a^	7.71 (2.10)^b^	1.74 (1.02)^c^	<.0001
Access time (min)	2.63 (0.75)	2.44 (0.66)^b^	17.07 (6.81)^c^	<.0001
Thrombolytic time (d)	6.39 (1.50)^a^	6.83 (1.41)	7.65 (1.15)^c^	<.0001
Urokinase (U)	324.51 (68.10)^a^	357.94 (48.31)	375.63 (51.52)^c^	<.0001
Subsequent interventions	25 (49.0)	26 (76.5)	58 (66.6)	0.049
PTA	1 (2.0)	2 (5.9)	1 (1.1)	
PTA + Stent	24 (47.1)	24 (70.6)	57 (65.5)	

Data presented as mean (SD) or No. (%).

a ICVA vs IPVA, *P* < 0.05.

b IPVA vs CFVA, *p* < .05.

c CFVA vs ICVA, *p* < .05.

### Thrombolytic efficacy

Segmental angiographic analysis revealed that antegrade approaches (IPVA and ICVA) yielded vastly superior thrombus resolution within the femoral and popliteal segments compared to CFVA (Table 3). This enhanced distal clearance directly translated into markedly improved inflow patency for the IPVA (91.2%) and ICVA (100%) groups, standing in stark contrast to the CFVA group (44.8%, *P* < 0.001). Rates of deep femoral vein axial transformation and outflow patency did not differ significantly among the cohorts.

**Table 3:** Effective thrombolysis rate in each group.

Characteristics	ICVA (*N* = 51)	IPVA (*N* = 34)	CFVA (*N* = 87)	*p* Value
Popliteal vein	51 (100)	33 (97.1)^a^	65 (74.7)^b^	<.0001
Femoral vein	51 (100)	31 (91.2)^a^	40 (46.0)^b^	<.0001
Common femoral vein	44 (86.3)	28 (82.4)	71 (81.6)	0.772
External iliac vein	45 (88.2)	29 (85.3)	75 (86.2)	0.914
Common iliac vein	43 (84.3)	27 (79.4)	66 (75.9)	0.499
Inflow Patency	51 (100)	31 (91.2)^a^	39 (44.8)^b^	<.0001
Outflow Patency	42 (82.4)	26 (76.5)	61 (70.1)	0.270
Axial transformation of the deep femoral vein	1 (2.0)	3 (8.8)	5 (5.7)	0.362

Data presented as mean (SD) or No. (%).

a IPVA vs CFVA, *p* < .05.

b CFVA vs ICVA, *p* < .05.

### Safety outcomes

Regarding procedural safety, the IPVA group recorded zero access-related complications. In contrast, while minor bleeding events were comparable across groups (Table 4), the ICVA approach was associated with a higher incidence of local adverse events (21.6%), including transient paresthesia and minor venous injuries, reflecting the anatomical complexity of calf vein puncture.

**Table 4:** Bleeding events.

Minor events	ICVA (*N* = 51)	IPVA (*N* = 34)	CFVA (*N* = 87)
Gross hematuria	0	1	0
Gingival bleeding	0	0	1
Skin ecchymosis	1	0	0
Hematoma	0	1	1

Data presented as number.

### Long-term clinical outcomes

**Figure 1:**
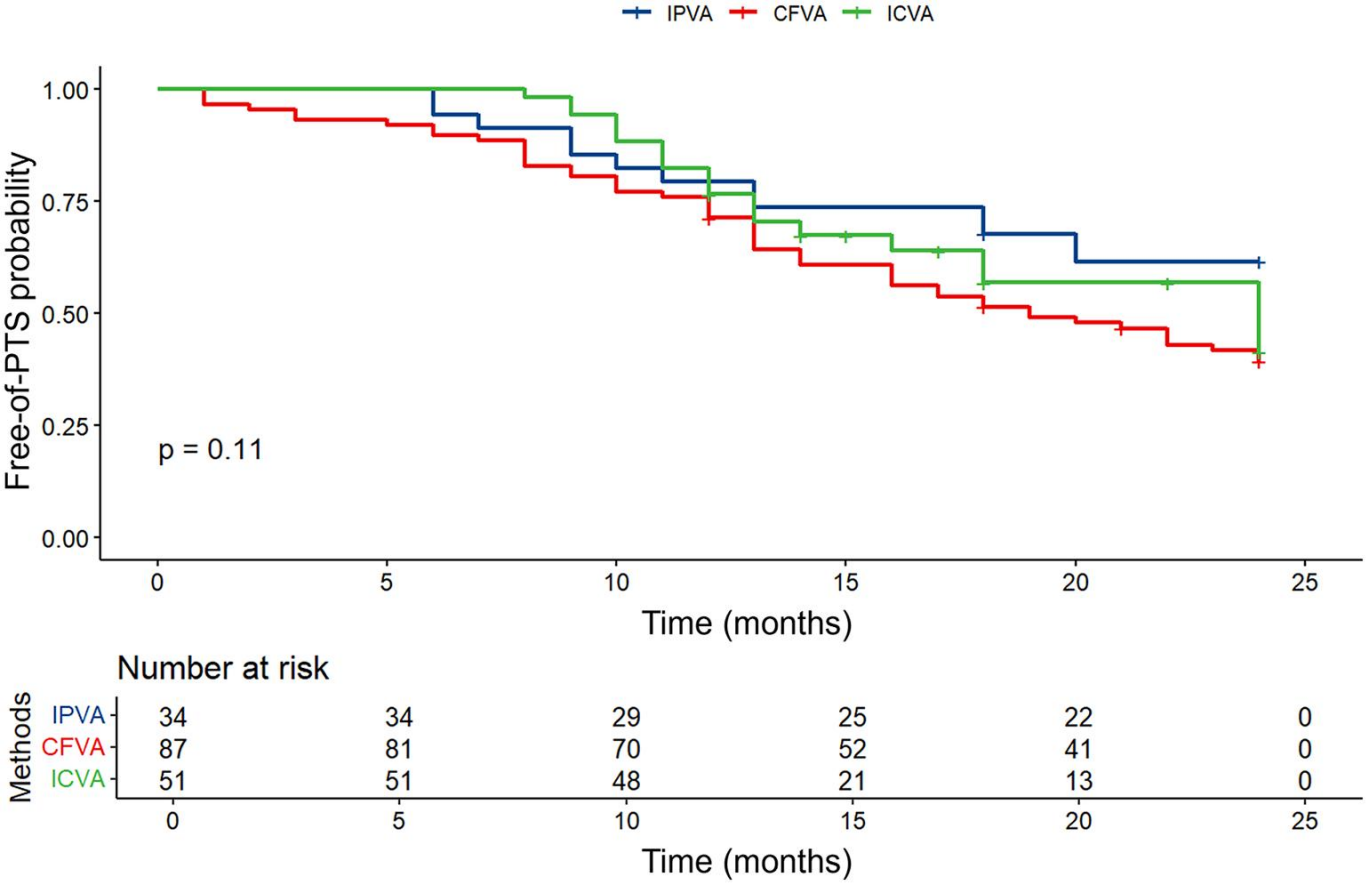
Freedom from post-thrombotic syndrome (PTS) over two years. The freedom from PTS among the three groups by Kaplan–Meier analysis showed no significant difference (*P* = 0.11). CFVA, the contralateral femoral venous access; IPVA, ipsilateral popliteal venous access; ICVA, ipsilateral calf venous access.

At the 2-year clinical landmark, the incidence of PTS was significantly higher in the CFVA group (59.8%) than in the IPVA (38.2%) and ICVA (41.2%) cohorts (*P* = 0.033) (Table 5). However, PTS severity and comprehensive quality-of-life assessments (VEINES-QOL/Sym) demonstrated no significant between-group differences. Correspondingly, Kaplan–Meier analysis across the extended follow-up period indicated that the overall PTS-free survival trajectory did not reach statistical significance (Log-rank *P* = 0.11) (Figure 1).

**Table 5:** Follow up.

Characteristics	ICVA (*N* = 51)	IPVA (*N* = 34)	CFVA (*N* = 87)	*p* Value
PTS	21 (41.2)	13 (38.2)	52 (59.8)	0.033*
PTS severity				0.107
Mild	11 (21.6)	8 (23.5)	25 (28.7)	
Moderate	10 (19.6)	3 (8.8)	21 (24.1)	
Severe	0	2 (5.9)	6 (6.9)	
Quality of life				
VEINES-QOL	54.22 (3.73)	54.59 (4.23)	53.08 (5.02)	0.168
VEINES-Sym	52.49 (3.81)	53.35 (4.22)	51.62(4.99)	0.149

Data presented as mean (SD) or No. (%).

*The incidence of PTS among CFVA group, IPVA group and ICVA group showed statistical significance.

### Factors associated with PTS

**Figure 2:**
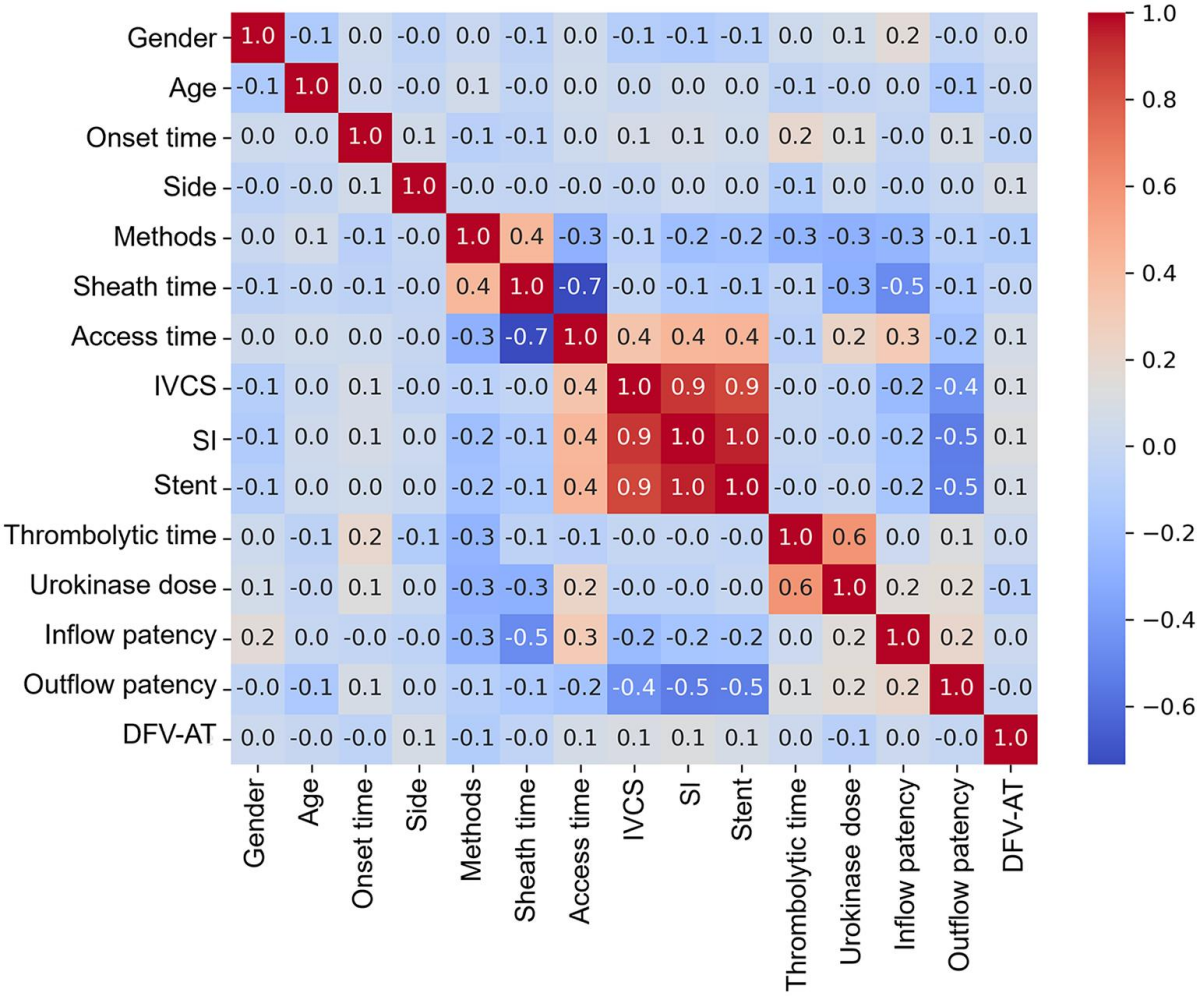
Interrelationships among the factors. A heatmap was employed to visually represent the correlation matrix of 15 factors, effectively illustrating the strength and direction of the relationships between each pair of factors. IVCS, iliac vein compression syndrome; DFV-AT, the axial transformation of the deep femoral vein; SI, subsequent interventions.

**Figure 3:**
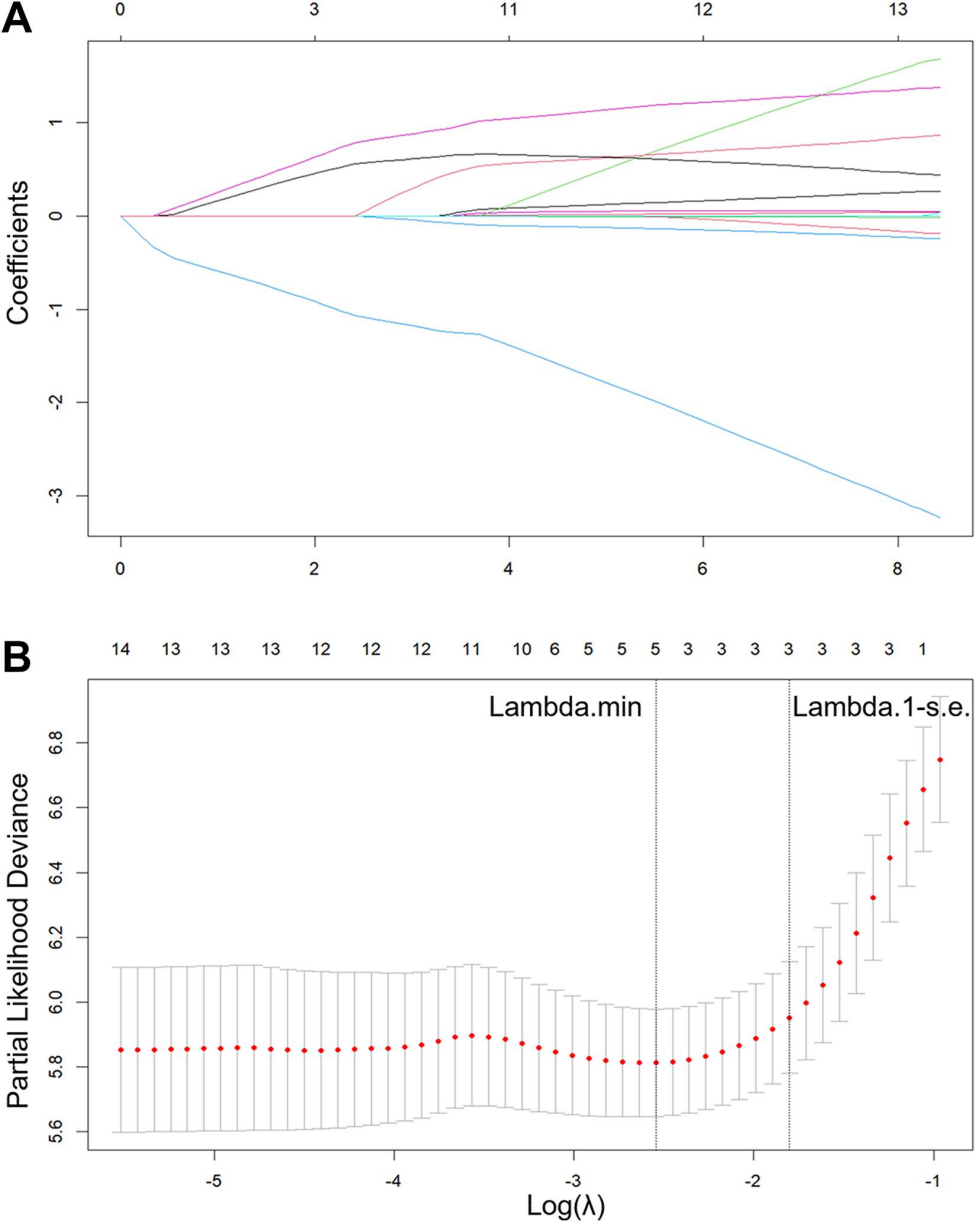
LASSO analysis for feature selection. LASSO coefficient profiles of 15 factors **(A)**. Five risk factors selected using LASSO Cox regression analysis. The two dotted vertical lines were drawn at the optimal scores by minimum (0.078) and 1-s.e. (0.165) criteria **(B)**.

**Figure 4:**
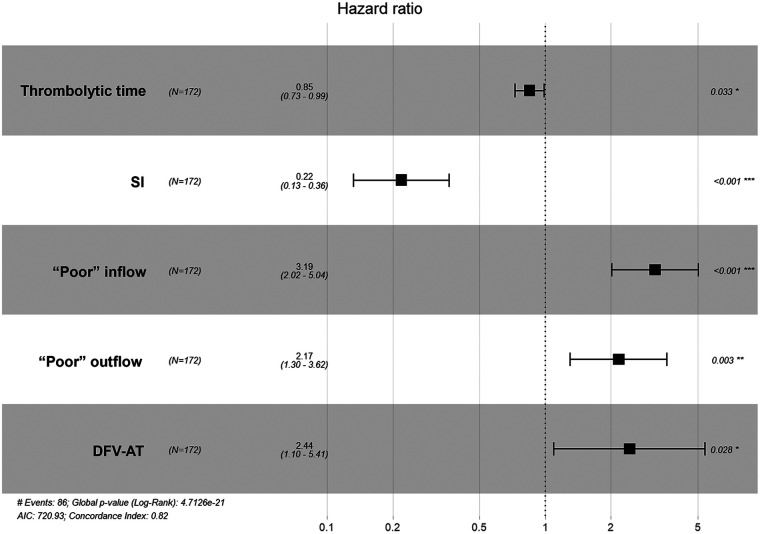
Five factors associated with post-thrombotic syndrome (PTS). The forest map showed the five factors associated with PTS by Cox multivariate analysis. DFV-AT, the axial transformation of the deep femoral vein; SI, subsequent interventions.

**Figure 5:**
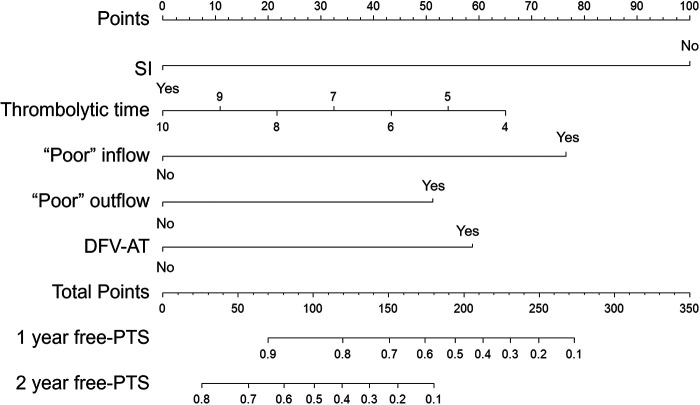
Nomogram for one- and two-years free-of-PTS. The nomogram was valued to obtain the probability of one- and two-years free-of-PTS by adding up the points identified on the points scale for each variable (including thrombolytic time, subsequent interventions, inflow patency, outflow patency). DFV-AT, axial transformation of the deep femoral vein; SI, subsequent interventions.

Given the pronounced multicollinearity observed among the fifteen clinical and procedural variables via Heatmap analysis (Figure 2), a LASSO-Cox regression model was applied to rigorously select prognostic features (Figure 3). Multivariate Cox analysis subsequently confirmed that prolonged thrombolysis duration (HR 0.85; 95% CI 0.73–0.99; *P* = 0.033) and the utilization of subsequent endovascular interventions (HR 0.22; 95% CI 0.13–0.36; *P* < 0.001) served as independent protective factors. Conversely, poor inflow patency (HR 3.19; 95% CI 2.02–5.04; *P* < 0.001), poor outflow patency (HR 2.17; 95% CI 1.30–3.62; *P* = 0.003), and the presence of deep femoral vein axial transformation (HR 2.44; 95% CI 1.10–5.41; *P* = 0.028) robustly predicted increased PTS risk (Figure 4). Synthesizing these independent drivers, we developed a prognostic nomogram that demonstrated an excellent discriminative capacity (C-index = 0.82; 95% CI 0.78–0.86) for predicting 1- and 2-year PTS-free probabilities (Figure 5).

## Discussion

Our study demonstrates that antegrade CDT access approaches (IPVA and ICVA) significantly enhance femoropopliteal thrombus dissolution and inflow patency compared to the traditional CFVA route, while concurrently reducing both the required thrombolysis duration and urokinase dosage. Furthermore, the identification of key prognostic factors—specifically, venous patency, deep femoral vein axial transformation, and endovascular optimization—highlights that long-term PTS prevention relies on comprehensive hemodynamic restoration.

Over the past decade, the incidence of venous thromboembolism has surged in China (20). While CDT is widely utilized—with over 85% of proximal DVT cases undergoing catheter-directed interventions (15)—standardized protocols for complex morphologies remain elusive (21). The CaVenT study robustly supported the “open-vein” hypothesis, demonstrating that successful venous recanalization via CDT translates to reduced PTS risk and improved quality of life (4). While these findings popularized the popliteal approach for standard proximal DVT, managing “entire-limb DVT”—which encompasses massive thrombus burdens across both the iliofemoral and femoropopliteal segments—presents unique technical and hemodynamic challenges.

Access site selection is the fundamental first step in addressing these challenges. CFVA is often favored for its straightforward puncture, reflected in our cohort's remarkably short sheath insertion time (1.74 ± 1.02 min). However, negotiating the anatomical complexities at the IVC-iliac junction (frequently exacerbated by underlying iliac vein compression) often prolongs the overall access establishment time. This is consistent with Li et al., who reported extended fluoroscopy and operation times for CFVA in acute iliofemoral DVT (22), mirroring our finding of a 17.07 ± 6.81 min access duration for the CFVA group.

Conversely, antegrade strategies (IPVA and ICVA) offer distinct procedural profiles. The IPVA approach demonstrated a moderate sheath insertion time (7.71 ± 2.10 min) with zero access-related complications, benefiting from the popliteal vein's larger caliber and superficial trajectory. ICVA, while theoretically ideal for complete antegrade coverage, recorded the longest sheath insertion time (9.02 ± 2.88 min) and the highest complication rate (21.6%). This underscores the technical demanding nature of traversing small-caliber, anatomically complex calf veins (14, 23), suggesting that routine ultrasound guidance is mandatory to optimize ICVA safety, as advocated by Wang et al. (24).

Beyond procedural times, the primary divergence among these approaches lies in their respective pharmacokinetic and hydrodynamic efficiencies. Our segmental analysis revealed that CFVA yielded inferior thrombus clearance in the femoral and popliteal segments. This is likely attributable to retrograde infusion mechanics: thrombolytic agents tend to follow the path of least resistance, preferentially escaping through proximal catheter side-holes into the larger iliac veins, thereby depriving the distal thrombus of effective drug exposure. In contrast, IPVA and ICVA leverage the natural antegrade venous flow vector, maximizing physical drug-thrombus interaction in the distal segments. Interestingly, Ni et al. reported contrasting results, suggesting CFVA was superior for femoropopliteal clearance (11). However, their study utilized pharmacomechanical CDT (PCDT). We hypothesize that PCDT rapidly decompresses the local venous segment, altering regional pressure gradients and thereby mechanically facilitating the distal delivery of thrombolytic agents. In standard CDT, without such mechanical decompression, aligning the infusion vector with the physiological antegrade flow (via IPVA/ICVA) proves crucial for distal efficacy.

Notably, while the CFVA group exhibited a higher 2-year PTS incidence, the overall PTS-free survival across the extended follow-up did not differ significantly among the groups. This nuanced finding suggests that the initial access approach, while technically crucial, is not the sole determinant of long-term outcomes. To address the substantial multicollinearity among the complex clinical variables and uncover the true drivers of PTS, we introduced a LASSO-Cox regression model (25).

Our model identified poor inflow/outflow patency and deep femoral vein axial transformation as robust, independent risk factors for PTS. From a hemodynamic perspective, deep femoral vein axial transformation essentially represents a passive, non-physiological compensatory mechanism secondary to the prolonged occlusion of the superficial femoral vein (the primary inflow tract). When standard CDT approaches fail to achieve adequate distal thrombus dissolution, the deep femoral vein is forced to dilate to accommodate the compromised venous return. This persistent collateral dependency fails to fully alleviate ambulatory venous hypertension, thereby accelerating PTS progression. Conversely, our model highlighted that prolonged thrombolysis duration and subsequent endovascular interventions (e.g., stenting of residual iliac lesions) served as protective factors. These findings strongly align with the CaVenT study's emphasis on venous patency and underscore that comprehensive endovascular optimization—aiming to thoroughly restore the primary anatomical venous axis—is paramount. By integrating these key hemodynamic and procedural predictors, our developed nomogram offers a tangible, evidence-based tool to assist clinicians in individualized risk stratification and tailored management for entire-limb DVT.

This study has several limitations that should be acknowledged. First, its single-center retrospective design is inherently susceptible to selection bias, and the choice of vascular access was partially influenced by the operators' judgment of individual venous anatomy. Second, we utilized a low-dose, long-duration urokinase protocol rather than tissue plasminogen activator (tPA). While urokinase possesses lower thrombolytic potency, this regimen was strictly implemented in accordance with the Chinese expert consensus on standard interventional therapy for DVT, aiming to maximize patient safety and minimize hemorrhagic complications in our specific population (16). Importantly, because this standardized pharmacological protocol was uniformly applied across all three cohorts, it is unlikely to confound our primary comparative analysis of access-related outcomes. Third, our retrospective design inherently introduces selection bias in vascular access. While antegrade approaches (IPVA/ICVA) are theoretically preferred, the retrograde CFVA route was pragmatically chosen for patients with severe ipsilateral lower limb edema, or when distal veins exhibited stenosis, tortuosity, or variations precluding safe cannulation. Consequently, this bias may confound the direct comparison of technical efficacy and long-term patency across groups. Finally, while our developed nomogram demonstrated strong discriminative performance internally, it requires external validation in large-scale, multicenter prospective trials to confirm its broader clinical utility.

## Conclusion

For patients with acute entire-limb DVT, antegrade approaches (IPVA and ICVA) provide superior femoropopliteal thrombus resolution and enhanced inflow patency compared to the traditional CFVA route. While the choice of access site significantly influences early procedural success, the long-term risk of PTS is fundamentally driven by the comprehensive restoration of venous hemodynamics—specifically, inflow/outflow patency and the mitigation of deep femoral vein axial transformation. By integrating these critical variables, our proposed nomogram serves as a valuable, evidence-based tool to guide individualized risk stratification and optimize endovascular management strategies.

## Data Availability

The raw data supporting the conclusions of this article will be made available by the authors without undue reservation.
